# On Improving Wear Resistance of Cr-Al-N Coatings Using Dynamic Glancing Angle DC Magnetron Sputtering

**DOI:** 10.3390/nano11092187

**Published:** 2021-08-26

**Authors:** Pedro Renato Tavares Avila, Raíra Chefer Apolinário, Alisson Mendes Rodrigues, Jucielle Veras Fernandes, Romualdo Rodrigues Menezes, Gelmires de Araújo Neves, Haroldo Cavalcanti Pinto

**Affiliations:** 1 São Carlos School of Engineering (EESC), University of São Paulo, São Carlos 13563-120, SP, Brazil; pedrorenatoavila@gmail.com (P.R.T.A.); raira.apolinario@usp.br (R.C.A.); 2 Laboratory of Materials Technology (LTM), Federal University of Campina Grande, Av. Aprígio Veloso-882, Bodocongó, Campina Grande 58429-900, PB, Brazil; jucielle_fernandes@hotmail.com (J.V.F.); romualdo.menezes@ufcg.edu.br (R.R.M.); gelmires.neves@ufcg.edu.br (G.d.A.N.)

**Keywords:** wear protection, Cr-Al-N, DGLAD, dcMS

## Abstract

The development of alternatives for wear protection in surface engineering can be responsible for a significant decrease in energy waste as a large amount of the energy produced in the world is lost due to tribological contact. Dynamic Glancing Angle Deposition has been recently evaluated as a route to produce coatings with improved wear performance. In this technique, the substrate oscillates along with a determined range in front of the sputtering target during the growth of the film. In this study, five oscillatory ranges (0, ±5°, ±10°, ±15°, ±20°) were probed to manufacture nanostructured Cr-Al-N coatings using direct current magnetron sputtering, and their impact was investigated on the grain morphology, phase formation, chemical composition, and performance of the coatings. FEG-SEM revealed the formation of multilayer-like architecture across the grains of the coatings. The deposition rate and hardness improved, and a more than 2-fold decrease in the material loss was observed in a comparison between the stationary-deposited conventional coating and the sample produced under ±10° oscillatory range. This indicated the potential use of this technique in future surface engineering applications.

## 1. Introduction

Energy generation and consumption have been some of the main concerns in modern society within recent decades since non-renewable resources have been used at a rate that will bring them to scarcity in the next century. Along with the consumption, the pollution generated using these resources is associated with climate change perspectives for the coming decades. One of the alternatives to mitigate these effects is to enhance the efficiency of energy consumption. Overcoming friction in day-to-day activities is known to consume an appreciable amount of energy [1,2]. It is estimated that up to 23% of the global energy generated is lost due to tribological contact [3]. In this scenario, surface engineering plays a crucial role in designing components with improved tribological performance and enhancing energy usage efficiency.

Transition metal nitrides have been widely studied in recent decades as candidates for coatings applied to metal surfaces to provide protection in severe friction and wear applications in diverse industrial branches, e.g., machining, automotive, aerospace, bioengineering and others [4,5,6,7,8]. One of the nitride systems studied is the Cr-Al-N, with interesting mechanical properties, good corrosion resistance and high thermal stability, making it an important candidate when designing conjugated systems in surface engineering [9,10,11].

Several production routes are available to create thin films. Magnetron Sputtering (MS) is important in manufacturing hard coatings producing dense and well-adhered films [6,12]. Along with the materials and techniques chosen for the manufacture of coatings, deposition geometry is also an important factor. The deposition of nanostructured films using Glancing Angle Deposition (GLAD) has been recently proposed in the literature [13,14,15,16,17,18]. In this type of deposition, the substrate is placed at an oblique angle to the sputtering target so that the flux of arriving species is off-normal with relation to the substrate surface [16]. This geometry propitiates the formation of films with high porosity due to the shadowing effect and tilted columnar grains [19]. If the substrate is spun around its normal axis, the columnar shape of the grains can be changed to a variety of sculptured formats, e.g., helix, zigzag, etc. [16]. Several authors reported changes in properties related to these grains sculptures that suggest applications in fields as optics, electronics and chemical catalysis [18,20,21,22].

Recently, studies indicated that zigzag grain morphologies produced by GLAD present changes in microstructure, such as texture and residual stresses that can also impact its mechanical properties [23,24]. A variation of GLAD in which the substrate is rocked in front of the target during deposition, continuously changing the angle of arrival of the sputtered material, has been proposed and received the designation of Dynamic Glancing Angle Deposition (DGLAD) [25,26]. This technique also produces coatings with nanosculpted grains, in the form of a zig-zag, for instance. The authors found correlations between these structures and variations on hardness and texture of the coatings [27], as well as misorientation gradients and the zigzag grains corresponding to the oscillatory motion of the substrate [25,28,29]. This showed a potentiality in tailoring the mechanical performance of hard coatings by engineering the sculpture of their grains.

Despite the studies published addressing the nanostructured sculped coatings, their properties, and mechanical characteristics, to our knowledge, none have evaluated the effect of these features on the wear resistance. Furthermore, the potential of the DGLAD technique has not yet been entirely clarified due to a lack of research on the role of the magnitude of angular oscillation. If consistently proved, this technique will be available as a route to produce longer-lasting engineering surfaces and contribute to mitigating energy waste on friction. This work aims to understand the potentials of such a technique by systematically varying the range of oscillation used during DGLAD and observing it in the changes in microstructure and wear bearing capacity of coatings of Cr-Al-N produced by direct current Magnetron Sputtering (dcMS).

## 2. Materials and Methods

A total of 304 AISI stainless steel substrates were metallographically prepared using abrasive SiC sandpaper with progressive grits up to #2000 and polished using 6, 3 and 1 µm diamond suspensions and colloidal silica to achieve a mirror finish. Before deposition, the substrates were cleaned in an ultrasonic bath with acetone and blow-dried. All depositions were conducted in a HiPIMS 250 Physical Vapor Deposition (PVD) chamber (PLASMA-LIITS, Campinas, Brazil). The substrates were mounted 65 mm apart from the targets. The sample holder is part of a carousel that can perform oscillatory movements rocking the sample in front of the target surface in a predetermined angular range and period. This particular setup has been described in detail in previous publications and is responsible for creating the DGLAD motion [25,28,29]. By rocking the substrate, the angle of incidence of the flux from the target (zenith) is continuously varied between the angular limits established and at a speed corresponding to the period of duration of the oscillations. Both the period of duration and the angular limitations can be chosen by the operator. In this paper, the period of duration of the oscillation was kept unchanged for all samples (30 s), and the oscillatory range was systematically varied for investigation (0, ±5°, ±10°, ±15°, ±20°).

The depositions were carried out in an atmosphere of N_2_ and Ar with a flow rate of 50 and 40 sccm, respectively, and pressure of 0.266 Pa at 400 °C. A Pinnacle MDX (Advanced Energy, Fort Collins, CO, USA) direct current (DC) power supply was used to provide −180 V substrate bias tension. The depositions were made using another Pinnacle MDX power supply with 900 W of power. CrAl 30/70 at % targets with a purity of 99.5% were utilized.

Before the deposition, all samples were ion etched for 1 h using Cr+ ions provided by HiPIMS discharges with 600 W average power, 104 Hz of pulse frequency, 50 µs of ton and −800 V to clean the substrate’s surface from residual contamination and promote ionic implantation to improve adhesion between the coating and the steel. A base layer of metallic Cr was deposited before the Cr-Al-N layer in all samples with about 10% of the total thickness of the coating.

Morphology and thickness of the coatings were assessed using Scanning Electron Microscopy (SEM) using a FEG-Inspect F-50 microscope (FEI, Eindhoven, The Netherlands). Roughness measurements and surface maps were obtained by Atomic Force Microscopy (AFM) using a NanosurfFlex (Nanosurf, Liestal, Switzerland) in tapping mode.

Energy Dispersive Spectroscopy (EDS) analyses using a silicon drift windowless Apollo X SDD detector from EDAX (Mahwah, NJ, USA). The measurements were calibrated using a sample of Cr-Al-N of known composition as reference.

X-ray Diffraction (XRD) was performed in θ–2θ geometry using a Panalytical MRD-XL (Panalytical, Almelo, The Netherlands) with Mo Kα radiation (0.7093 Å).

Nanoindentation tests were performed using a PB1000 mechanical tester (Nanovea, Irvine, CA, USA) with a load of 50 mN. Seven different indentations were made for each sample with a diamond Berkovich tip, and the mean standard deviation was calculated. Hardness and Elastic modulus were calculated using the Oliver–Pharr relation [30].

Linear reciprocating sliding wear tests were performed using the same PB1000 mechanical tester according to the ASTM G113 standard. The tests were performed at 23 °C with no lubrication. Al_2_O_3_ spheres of 6 mm in diameter were chosen as counter-body. The normal load applied was 10 N. Mean sliding speed was 60 mm/s alternating in a reciprocating motion and to a track of 3 mm of length for 200 cycles, totalizing a sliding distance of 1200 mm.

Wear in the coatings on the surfaces of the Al_2_O_3_ spheres was visualized using a non-contact optical profilometer. ASTM G133-05 standard was followed to calculate wear volume and wear rate. Six cross-section profiles from the trench of each sample were extracted. Their area was calculated to obtain a mean area of loss material multiplied by the total sliding distance to determine the average wear volume.

## 3. Results

### 3.1. Morphology, Structure and Chemical Composition

**Figure 1:**
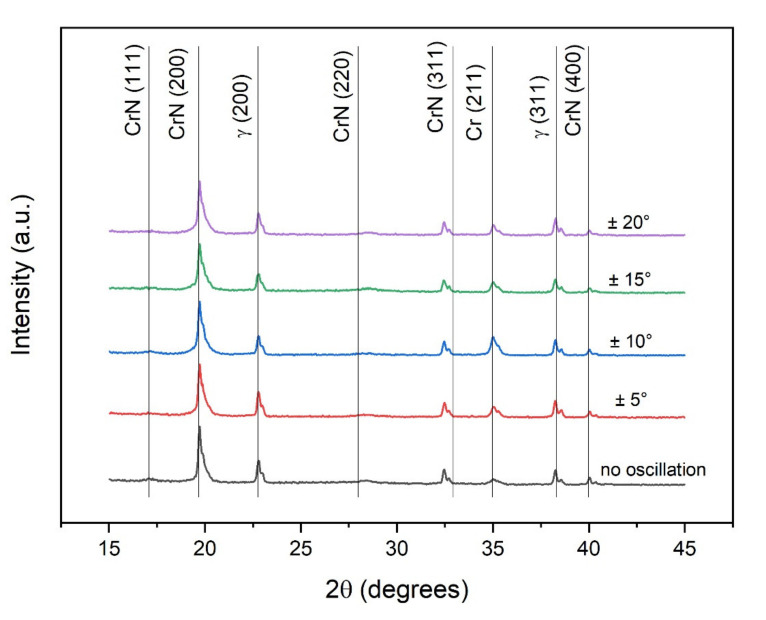
X-ray θ–2θ diffractograms of the dcMS Cr-Al-N coatings using DGLAD with different oscillatory ranges.

The XRD measurements for the coatings produced under the five different values of oscillatory range revealed peaks from the γ-steel substrate are visible along with Cr peaks from the base layer (as seen in Figure 1). Besides these two materials, only peaks from B1-CrN are detected regardless of the type of magnetron sputtering technique applied or of the oscillatory range of the substrate during deposition. There is no expressive change in texture related to the oscillatory range of the substrate.

The (200) is the preferential orientation for all coatings, which is expected for Cr-Al-N in conditions of high adatom mobility [29,31].

**Figure 2:**
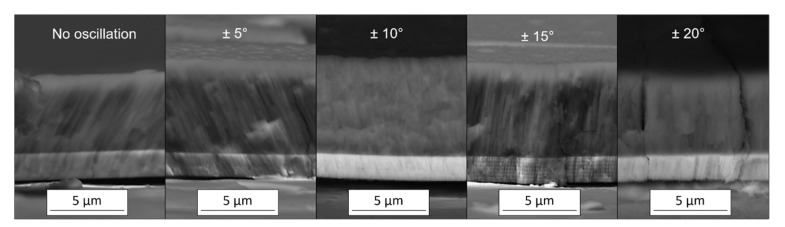
Cross-section SEM images of dcMS produced coatings (BSE mode).

Cross-sectional images of the dcMS coatings are presented in Figure 2. The brighter layer corresponds to the Cr metallic base layer. The coatings present a fiber morphology with columnar grains. An increase in the thickness of the Cr-Al-N coatings from the condition of no oscillation up to ±10°, along with a gradual decrease from ±10° to ±20°, is noticeable. Since all depositions were made using the same total time of 2 h, these results indicate that the deposition rate depends on the oscillatory range in DGLAD.

**Figure 3:**
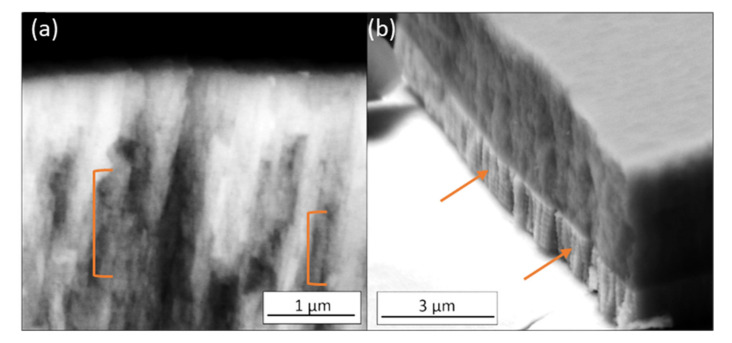
Coatings presenting the structure of the grains in detail. (**a**) ±15° and (**b**) ±20°. The effects of the DGLAD can be noticed as multilayer-like stripes across the grains (detailed by brackets and arrows).

The effect of the DGLAD oscillatory motion is most visible in the base layer of the ±15° and ±20° coatings in the form of multilayer-like nanostructures or corrugated grains, as evidenced in other studies [26,28]. A detailed view of those structures in higher magnification is provided in Figure 3. Examples of nanostructures are highlighted in brackets and arrows. The corrugated grains are more visible in the metallic base layer due to their higher deposition rate than the Cr-Al-N ceramic film. Higher deposition rates yield thicker corrugated layers. The periodicity of the nanostructures for the base layer is around 250 nm, which is consistent with the 30 s period of oscillation used in all depositions.

**Figure 4:**
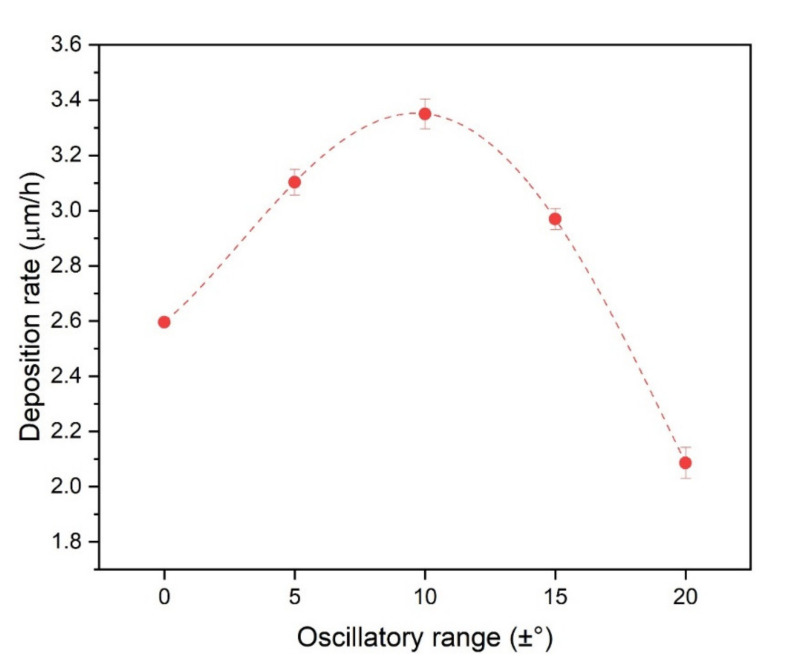
Deposition rate as a function of oscillatory range.

The deposition rate for the different oscillation conditions in Figure 4 indicates an expressive deposition rate maximum at the ±10° oscillatory range, corresponding to an increase of about 1.3 times compared to the no oscillation condition. The deposition rate is a fundamental aspect of coatings in industrial applications since a higher deposition rate usually indicates lower production costs [32].

One possible explanation for the dependence of deposition rate on oscillatory range takes into consideration that: (i) the region of a high density of the plasma corresponds not to a point but to an area in front of the target that is typically wider than the substrate dimensions in the shape of the so-called racetrack, and (ii) sputtering is a line-of-sight process. Therefore, the rocking oscillatory motion of the substrate during DGLAD provides better utilization of the whole sputtering cloud. This is the cause of the progressively thicker coatings observed in Figure 2 when the oscillatory range increases from 0 to ±10°. On the other hand, if the substrate oscillates in a too wide range, it gets out of the target’s line-of-sight for a short period and causes the film to stop growing temporarily, as is the case of the ±15° and ±20° in Figure 2 and the process experiences a drop-in deposition rate.

**Figure 5:**
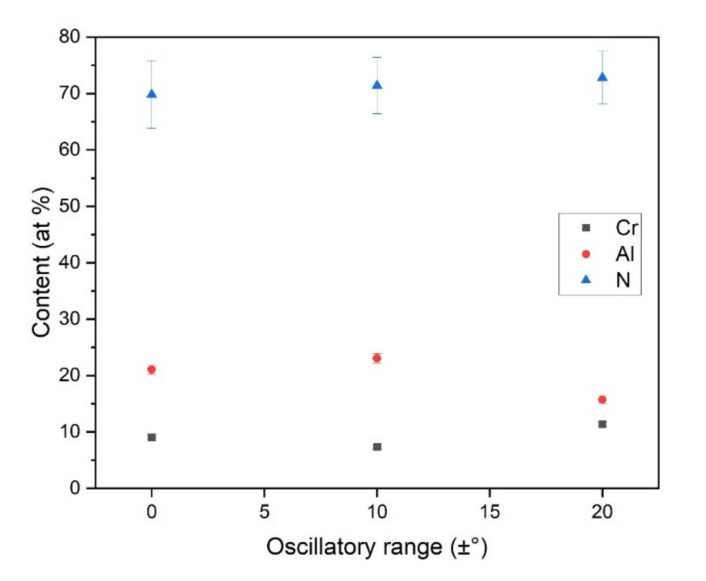
Chemical composition of the coatings produced under different oscillatory ranges.

The results of EDS measurements for the conditions of no oscillation, ±10° and ±20° samples are plotted in Figure 5. It is evident that the coatings presented high N content because of target poisoning and the low sputtering yield of Al-containing targets in the reactive atmosphere [29]. There is no observable systematic dependence of the chemical composition on the oscillatory range.

**Figure 6:**
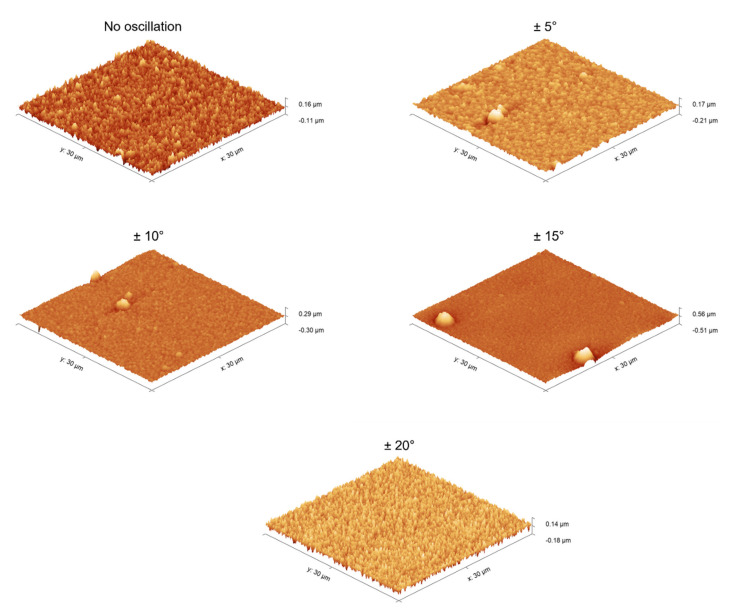
Surface morphology maps of the coatings obtained by tapping-AFM.

The coatings produced using direct current presented facets and an almost defect-free surface, with a small concentration of cone-shaped growth defects (e.g., in samples ±10° and ±15°), as seen in the surface maps in Figure 6 from the data collected by tapping-AFM. These defects are associated with flakes and impurity particles that attach to the substrate or the growing film surface in the early stages of the deposition. Frequently, the defects originated from the chamber walls (produced during thermal expansion of the component, for instance) and from residues of the cleaning process of the substrate surface [33].

The mean arithmetic (Ra), as well as the root, mean squared roughness (Rrms) values do not seem to vary systematically concerning the oscillatory range (see Table 1). Rsk, or skewness roughness, can be interpreted as the asymmetry of the amplitude distribution function, i.e., a positive value of Rsk indicates a prevalence of peaks while a negative Rsk corresponds to a predominance of valleys in the amplitude distribution function [34]. Therefore, the higher positive values of Rsk of the ±10 and ±15 coatings are another indication of the higher concentration of morphological defects on the surface.

### 3.2. Mechanical and Wear Performance

**Figure 7:**
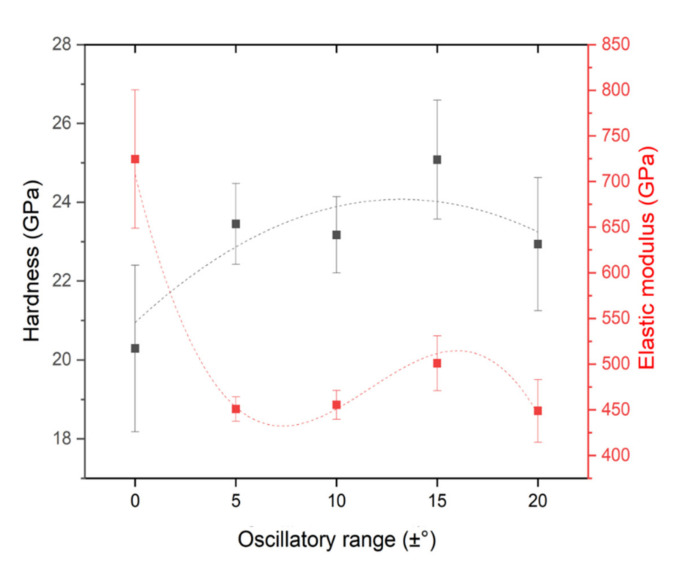
Hardness and reduced elastic modulus as functions of the oscillatory range.

The results of hardness and reduced elastic modulus measured by instrumented nanoindentation are presented in Figure 7. A dependence of hardness on the oscillatory range is visible as the DGLAD conditions are altered from no oscillation to ±5°, hardness tends to increase, being ±15° the harder condition (about 25 GPa). These results are associated with the corrugated nanostructures formed by substrate oscillation since no texture, or chemical composition changes are evident. From Figure 7, it can be seen that the ±20° condition presented inferior properties when compared to the ±15° specimen. This is attributed to the effects of oblique deposition at higher angles. Since it is responsible for shadow effects that can produce less dense and well-adhered coatings [20], it can deteriorate some of the gains obtained by the multilayer-like architecture of the DGLAD.

**Figure 8:**
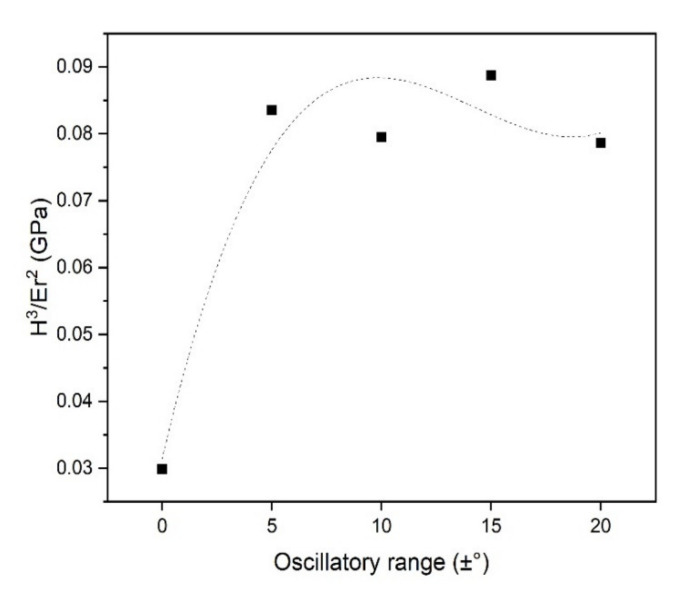
H^3^/Er^2^ as a function of the oscillatory range of coatings produced using dcMS.

Hardness and reduced elastic modulus (Er) are two of the relevant mechanical properties easily measurable by using nanoindentation tests and can indicate if the response of the coating will be through elastic or plastic mechanisms. The ratio H^3^/Er^2^ is described as the resistance to plastic deformation, being higher values of H^3^/Er^2^ indicate higher toughness, i.e., lower energy dissipation to permanent deformation [35,36]. The values of H^3^/Er^2^ in Figure 8 indicate an increasing trend in resistance to plastic deformation with an increase in oscillatory range as an effect of the expressive dependence of the hardness on the oscillatory range.

**Figure 9:**
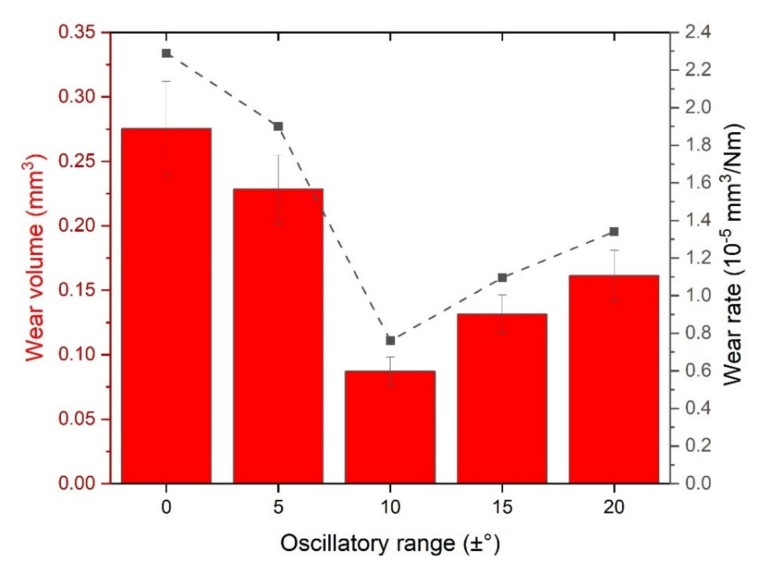
Effect of the oscillatory movement of the substrate on the volume loss and wear rate of the coatings under linear reciprocating tests.

The performance of the different dcMS coatings in the linear reciprocating test in terms of wear volume and wear rate (Figure 9) indicates that both features present a decreasing trend with increasing oscillatory range from 0 up to ±10°, and a tendency to increase for the conditions of ±15° and ±20°. A reduction in the worn volume of about three times is evident from the condition of no oscillation to the condition of ±10°.

**Figure 10:**
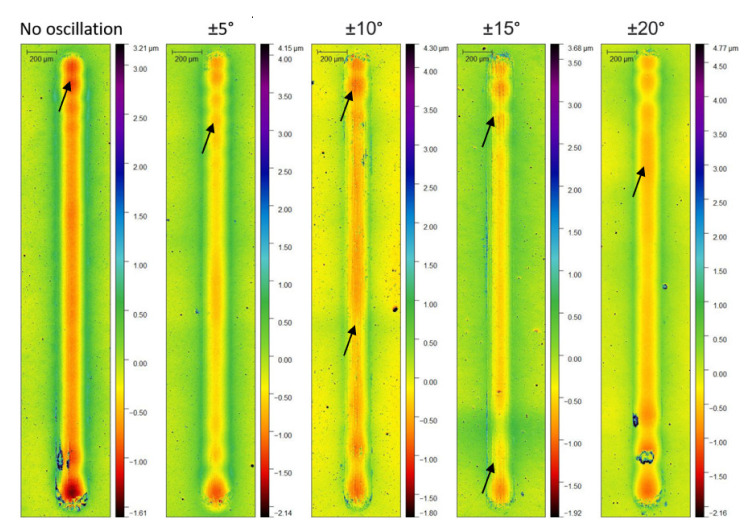
Wear tracks produced by the Linear Reciprocating Wear test on the surface of the coatings.

All the wear tracks generated from the linear reciprocating wear tests, as measured by optical profilometry, presented a pile-up of material at the edges (see Figure 10). Furthermore, grooves at the bottom of the tracks were visible, indicating the occurrence of abrasive wear (indicated by arrows).

**Figure 11:**
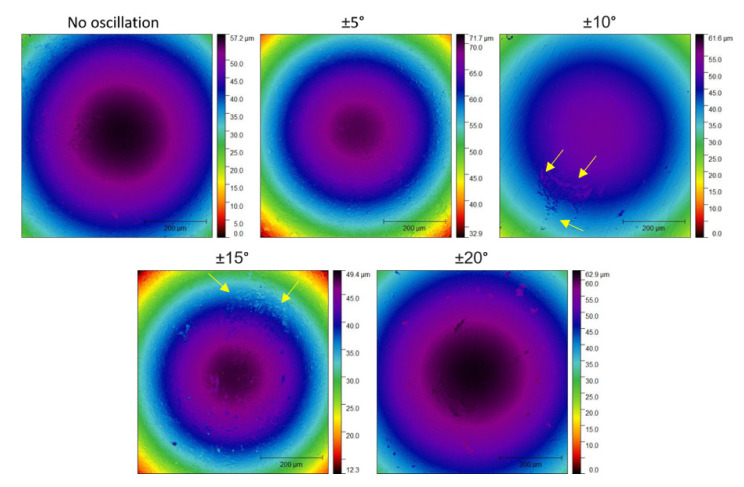
Wear of the Al_2_O_3_ sphere counter body slid against the dcMS coatings. Yellow arrows indicate the adhered material.

The wear signals on the counter body are also relevant and can help elucidate the wear mechanisms present on the system [37]. In Figure 11, the worn surface of the Al_2_O_3_ sphere presented an exact circular shape of wear marks indicating removal of a calotte of material along with the tests, as expected for abrasive wear mechanisms. The worn calottes follow the trend in wear rate from Figure 11 and decrease in radius with an increase of oscillatory range up to ±10° when they start to increase up to ±20°. The only exception to this was the sphere used in the test against the coating of ±10° and ±15° condition, which exhibited signs of material adhered to the surface.

**Figure 12:**
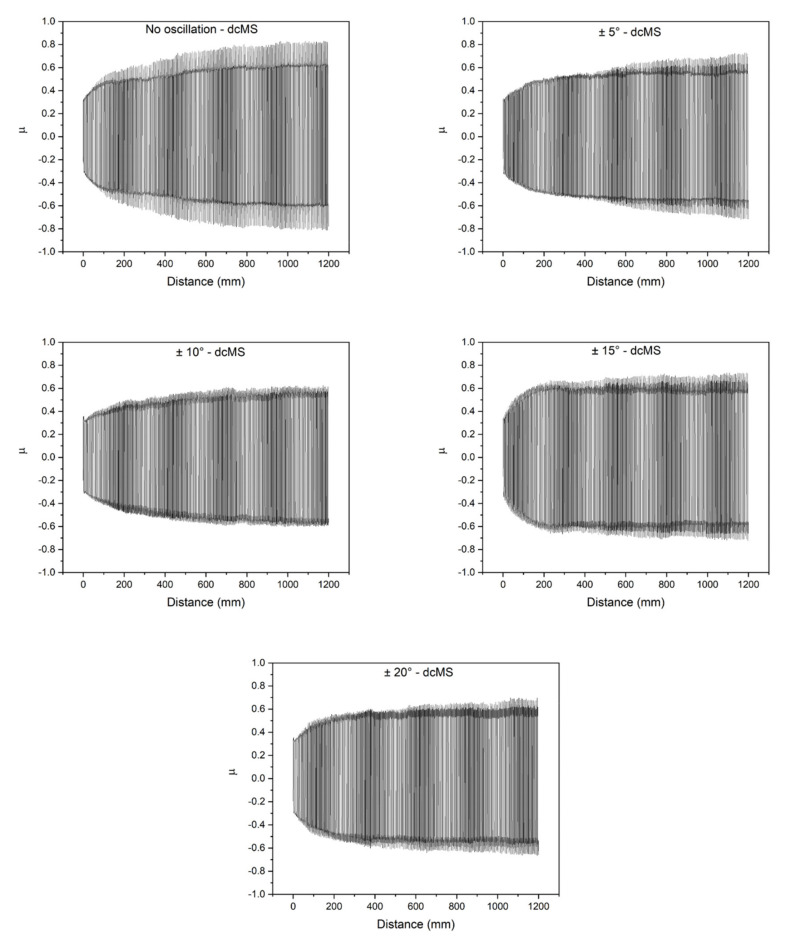
Coefficient of friction between the Al_2_O_3_ sphere and the dcMS coatings for different oscillatory ranges measured continuously along with the Linear Reciprocating Wear tests.

Another important feature for understanding the wear phenomena of a system and that is usually measured in linear reciprocating tests is the coefficient of friction (µ). The evolution of µ along the test for the coatings produced with different oscillatory ranges can be seen in Figure 12. The variation between positive and negative values of µ indicates the changes in the sliding direction. All samples presented a rapid and steep increase in µ in the first few cycles of the test. This is expected, as the asperities present on the surface of the coatings and on the sphere make contact and cause resistance to the sliding force, causing the coefficient of friction to increase. Once the asperities are removed, µ stabilizes. At the final part of the tests, a slower increase is observed, typically caused by debris formed from the wear and accumulated in the trenches [38].

The peak values of µ in both directions (µ_max_ and µ_min_) are presented in Table 2. Since the coefficient of friction measured in linear reciprocating wear tests is a sinusoidal function with different regimes, sometimes other values besides maximum and minimum are relevant. µ_rms_ stands for root mean square coefficient of friction and is an effective value for µ, a statistical measurement of the magnitude of its variation along with the test [39]. The values of µ_rms_ are also presented in Table 2. There is a positive relation between µ_rms_ and the wear rate calculated by the volume loss (Figure 9), being a minimum of µ_rms_ correspondent to the lower wear rate (±10° condition).

## 4. Discussion

The use of DGLAD during the production of the coatings produces grains with a multilayer-like nanostructure, visible in the detailed SEM images (Figure 3a,b). In previous studies from this research group and others, the corrugated architecture of the grains formed by the dynamic oblique deposition is associated with periodic variations in residual stresses and misorientation gradients [23,28]. These features act as barriers to plastic deformation. Moreover, a common failure mechanism for PVD coatings is through intergranular fractures that propagate along the columnar grains [40]. Since the oscillatory motion of the substrate produces films with corrugated grains, the propagation of intergranular cracks must follow its corrugated nature and constantly change its direction, losing an amount of energy each time [24]. Larger oscillatory ranges can accentuate this. These mechanisms enhance the hardness and decrease in stiffness with an increase of the oscillatory range since no other systematic change in microstructure or composition was detected.

Understanding the phenomena that drive the tribological performance of PVD coatings is complex and depends on several factors such as mechanical properties of the film, surface roughness and lubrication conditions. Hardness and reduced elastic modulus (Er) are two of the relevant mechanical properties easily measurable by using nanoindentation tests and can indicate if the response of the coating will be through elastic or plastic mechanisms. The improvement in wear performance of the samples produced under oscillatory motion compared to the static one can be related to the improvement in the H^3^/Er^2^ due to the multilayer-like structure and its mechanisms of energy dissipation discussed above. Recently, H^3^/Er^2^ (also referred to as resistance to plastic deformation or yield pressure) has been pointed as an accurate indicative measure of the wear performance of the material [41,42]. In the case of low H^3^/Er^2^ as for the dcMS sample produced under no oscillation condition, when put in sliding contact against a counter body, it can suffer brittle wear mechanisms due to its low toughness (low H^3^/Er^2^). This can also generate debris in the wear track that contributes to abrasion.

As the resistance to plastic deformation increases, so does the wear resistance. In the reciprocating test, an expressive reduction in material loss is observed with a progressive rise in oscillatory motion up to ±10°. Further, enhance of the oscillatory range is followed by a gradual increment in material removal. This indicates an optimum value for wear resistance (±10°) as a possible result of the compromise between the formation of the multilayer-like nanostructure and the deterioration of the mechanical properties due to the oblique deposition. While one creates mechanisms of energy dissipation that enhance hardness and contribute to reducing material loss, the latter can create less dense coatings due to the shadowing effect present in high-angle oblique deposited coatings. With the increase in the oscillatory range, the latter reduces the gains of the multilayer-like structure. It is essential to highlight that this optimal condition is the same that presented the highest deposition rate. Therefore, the adequate use of the DGLAD setup can produce improved wear bearing coatings in shorter deposition times when compared to the conventional stationary deposition strategies.

These high-performance features associated with high production are especially relevant considering the development of more sustainable materials, as engineering surfaces more resistant to wear are produced. This saves energy in maintenance processes and avoids the loss of efficiency of components and requires less time and energy to be produced.

More studies regarding the development of this technique are still necessary to thoroughly access the potentials and limitations of the DGLAD architecture, for instance, in combination with high-performance techniques, such as HiPIMS.

## 5. Conclusions

After producing and characterizing dcMS coatings using DGLAD with different oscillatory ranges (no oscillation, ±5°, ±10°, ±15 ° and ±20°), the following conclusions can be drawn:

- The variations in oscillatory range did not produce any detectable texture or chemical composition change. The deposition rate is dependent on the oscillatory range, being ±10°, the condition with the highest deposition rate.
- Hardness improvement from ~20 up to ~25 GPa was obtained using DGLAD as an effect of the multilayer-like structure formed due to oscillation.
- ±10° oscillatory range produced coatings with best wear performance with about three times less material removed when compared to coatings produced with no oscillation.
- DGLAD is presented as a promising route to manufacture multilayer-like coatings with improved wear resistance capable of reducing energy loss by friction.

## Figures and Tables

**Table 1:** Roughness values for coatings produced under different ranges of the oscillatory motion of the substrate. Data is presented in terms of Mean Arithmetic Roughness (Ra), Root Mean Square Roughness (Rrms) and Skewness (Rsk).

Oscillatory Range	Ra	Rrms	Rsk
No oscillation	20.98	26.55	0.27
±5	17.26	23.01	0.51
±10	14.76	20.74	1.23
±15	27.39	49.73	2.82
±20	26.62	33.36	0.11

**Table 2:** Coefficient of friction of the coatings produced using dcMS under several oscillatory ranges.

Oscillatory Range	µ_rms_	µ_max_	µ_min_
No oscillation	0.572	0.831	−0.813
±5	0.524	0.728	−0.719
±10	0.495	0.626	−0.604
±15	0.571	0.735	−0.723
±20	0.519	0.698	−0.665

## Data Availability

Data available in a publicly accessible repository since the main manuscript source was a Ph.D. thesis. That Ph.D. thesis is online at https://doi.org/10.11606/T.18.2021.tde-14062021-090721. The author of the Ph.D. thesis and your supervisor are co-authors of the manuscript.
